# Perceptual Load-Dependent Neural Correlates of Distractor Interference Inhibition

**DOI:** 10.1371/journal.pone.0014552

**Published:** 2011-01-18

**Authors:** Jiansong Xu, John Monterosso, Hedy Kober, Iris M. Balodis, Marc N. Potenza

**Affiliations:** 1 Department of Psychiatry, Yale University School of Medicine, New Haven, Connecticut, United States of America; 2 Department of Psychology, University of Southern California, Los Angeles, California, United States of America; 3 Child Study Center, Yale University School of Medicine, New Haven, Connecticut, United States of America; Macquarie University, Australia

## Abstract

**Background:**

The load theory of selective attention hypothesizes that distractor interference is suppressed after perceptual processing (i.e., in the later stage of central processing) at low perceptual load of the central task, but in the early stage of perceptual processing at high perceptual load. Consistently, studies on the neural correlates of attention have found a smaller distractor-related activation in the sensory cortex at high relative to low perceptual load. However, it is not clear whether the distractor-related activation in brain regions linked to later stages of central processing (e.g., in the frontostriatal circuits) is also smaller at high rather than low perceptual load, as might be predicted based on the load theory.

**Methodology/Principal Findings:**

We studied 24 healthy participants using functional magnetic resonance imaging (fMRI) during a visual target identification task with two perceptual loads (low vs. high). Participants showed distractor-related increases in activation in the midbrain, striatum, occipital and medial and lateral prefrontal cortices at low load, but distractor-related decreases in activation in the midbrain ventral tegmental area and substantia nigra (VTA/SN), striatum, thalamus, and extensive sensory cortices at high load.

**Conclusions:**

Multiple levels of central processing involving midbrain and frontostriatal circuits participate in suppressing distractor interference at either low or high perceptual load. For suppressing distractor interference, the processing of sensory inputs in both early and late stages of central processing are enhanced at low load but inhibited at high load.

## Introduction

Attention facilitates goal-directed behavior by focusing on targets and inhibiting interference from distractors. Several theories have been proposed to explain the brain mechanisms underlying inhibition of distractor interference. *Early selection theory* proposes that attention filters irrelevant information during perceptual processing [1], while *late selection theory* proposes that attention selects relevant information for response and/or memory storage after perceptual processing [2]–[4]. More recently, the *load theory of selective attention* proposed that the neural substrates underlying inhibition of distractor interference is dependent on the perceptual load of goal-directed tasks. It proposes that attention prevents distractor interference during perceptual processing at high perceptual load and after perceptual processing at low load [5]–[7].

When considering the load theory, one might pose the following hypotheses. First, the early stage (e.g., perceptual processing) of central processing will process distractors at low load because the spared processing resources from the central task will spill over to distractors. Second, the late stage (e.g., after perceptual processing) will actively differentiate perceived distractors from targets at low load. Third, the central processing passively excludes distractors from processing at high load because central tasks may consume all processing resources [5], [6], [8]. Because this theory hypothesizes that the brain actively processes distractors at low load more so than at high load, one might predict that both early and late stages of central processing will show a greater distractor-related activation at low relative to high load. Part of this prediction appears consistent with functional magnetic resonance imaging (fMRI) findings. For example, fMRI studies have demonstrated a greater distractor-related activation in visual and/or auditory cortex at low relative to high perceptual load, even when the distractors were not visible or in a different sensory modality from the targets [7], [9]–[15].

While fMRI studies consistently support the prediction of a greater distractor-related activation in the early stages of central processing at low relative to high load, there is little evidence supporting the prediction of a greater distractor-related activation in later stages at low relative to high load. Data suggest that the neural correlates of later stages of central processing involve frontostriatal circuits [6], [8]. Both human and animal studies indicate that frontostriatal circuits are involved in inhibiting distractor interference by filtering distractors, differentiating targets from distractors, and selecting appropriate responses [16]–[18]. One of the aforementioned fMRI studies reported that distractor-evoked activation in the inferior frontal gryus (IFG) was reduced at high relative to low perceptual load [15], seemingly consistent with above prediction. On the other hand, another study did not find significant differences in distractor-evoked activation in frontostriatal circuits between low and high loads [10], and did not appear to support the prediction of a greater distractor-related activation in the late stage at low relative to high load.

To investigate further the influence of perceptual load on distractor-evoked activation in the frontostriatal circuits, we assessed the whole-brain activity of 24 healthy participants using fMRI as they performed a novel visual target-identification task under varying load and distractor conditions. The task features two different perceptual loads (i.e., low and high load), and each load has two distractor conditions (i.e. with and without visual distractors). Based on the load theory, as well as the fMRI studies reviewed above, we predicted that distractors would be associated with a greater increase in activity not only in the occipital cortex, but also in frontostriatal regions at low relative to high load. We examined this prediction by comparing distractor-related changes in blood oxygen level dependent (BOLD) signal at different perceptual loads. Data confirming or refuting this prediction would not only have theoretical implications with respect to the validity of the load theory, but also provide empirical data on the neural mechanisms underlying selective attention in different environmental contexts.

## Materials and Methods

### Participants

Twenty-seven healthy adults from the community of the University of California Los Angeles (UCLA) gave written informed consent to participate in this study, which was approved by the Institutional Review Board at the UCLA. Data from three subjects were excluded from analysis because one subject fell asleep and the other two reported inadequate vision correction in the scanner. The final sample size included 24 participants (ages 23–41 years, all right handed, 12 females).

### Task Stimuli

**Figure 1:**
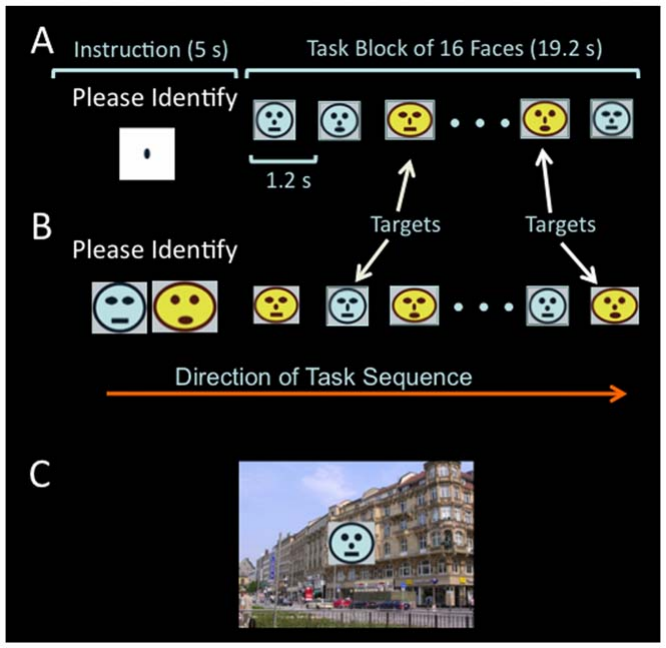
Diagram demonstrating elements of the visual target identification task. A) A block sequence in the *low perceptual load without distractor* condition. First, the instruction screen shows “Please Identify” above an elongated nose, for 5 seconds. The elongated nose informs the participants to identify targets in the next task block based on nose shape. During the task block, 16 faces are presented one at a time, for 100 ms each (with 1.1 second inter-stimulus interval). B) A block sequence in the *high perceptual load without distractor* condition. The instruction screen shows “Please Identify” above two faces without a nose, which informs the participants to identify targets matching the unique combination of face color, and shapes of face, eyes, and mouth in the next task block. C. An example of a face overlaid on a background distractor (i.e., scene picture). The same 64 scene pictures were used in task blocks of both low and high perceptual load. The same instruction screens for low and high perceptual load were used in the distractor and no-distractor conditions.

The task used 16 schematic faces as targets and 64 scene pictures as background distractors (Fig. 1). Because faces and scenes should specifically activate fusiform gyrus and parahippocampus gyrus, respectively [19], [20], using them as targets and distractors was anticipated to permit separation of target-related from distractor-related activations in the visual cortex. Faces were composed from different combinations of five facial features (shape of face, eyes, nose, and mouth, and face color). Each facial feature had two different forms (e.g., shapes: round and oval; colors: yellow and blue). No single face was completely unique in all facial features from other faces in the group, so that each face always shared one or more features with other faces. The round faces extended approximately 2° in diameter, and the oval faces extended approximately 1.6° in height and 2.2° in width. The scene pictures were presented as the background of the relevant stimuli (e.g., target and non-target faces; Fig. 1).

### Task Design

The task used a 2×2 factorial design with two perceptual loads (low, high) and two distractor conditions (with, without). At low load, any face with an elongated nose was a target; participants could simply search features to identify the target (Fig. 1A). At high load, targets were defined by face color, and shape of eyes, mouth, and face (Fig. 1B). Participants were required to search for these four features in conjunction to identify the target. Stimulus presentation was implemented with Psychtoolbox (http://psychtoolbox.org/PTB-2/osx.html) and displayed on the screen of a Macintosh laptop.

Stimuli for each condition were grouped into blocks with the same 16 faces used in every trial block. These 16 face images, either alone or overlaid on a distractor image, were presented one by one in random sequence within each block. Each image was presented once for 100 ms. The interstimulus interval was 1.1 s, and the duration of each block was 19.2 s (i.e., (100ms+1.1s)×16 = 19.2). The instruction, “Please identify,” was presented for 5 s before each trial block above an instruction image specifying the facial features for the targets in the following task block (Fig. 1). Participants were required to identify targets specified for each block and to respond by pressing a button as soon as possible after detecting a target. There were four targets randomly positioned in the presentation sequence in each block, and no two targets appeared on consecutive trials. The central task was to identify targets based on the instructions given before each block, and the relevant stimuli in this task were faces, including both targets and non-targets.

Prior to scanning, each participant was trained until he or she achieved three consecutive performances of the task each with error rates less than 50% for both commission and omission errors at high perceptual load. During scanning, each subject performed three functional runs using three different task scripts. No feedback on task performance was provided during the entire scanning period. Within each run, each blocked condition was repeated four times, and the whole run lasted 387.2s. The order of blocks was randomized within each run, and the order of runs was counterbalanced across subjects. Each condition within one run (i.e., 4 blocks) consisted of 64 stimuli (i.e., 16×4). In the distractor condition at each load level within each run, each stimulus had a different distractor, but the same 64 pictures were used for both low and high load conditions to avoid potential differences in brain activation induced by different physical stimuli between perceptual loads. Task performance was assessed using reaction time (RT), rate of omission error, and rate of commission error. It has been suggested that omission and commission errors reflect deficits in different central processes (i.e., attention and impulsivity, respectively) [21]–[24]. Therefore, we assessed each error type separately rather than utilize measures that group domains together in assessing task performance. The performance data were analyzed using SPSS 16. General linear model (GLM) was used to assess the main effects of task loads and distractors and two-way interactions between task loads and distractors. Paired t-tests were performed post-hoc if significant two-way interactions were detected.

### Imaging Data Acquisition and Analysis

Functional images were acquired using gradient-echo EPI scanning sequence (TR/TE = 1500/30 ms, Flip angle = 70°, 26 slices, 3 mm thick with 1.2 mm skip, 3.125×3.125 mm in plane pixels) with a Siemens Allegra 3T system. The scanning plane was off the AC-PC line rostrally at 20°. The thin scanning slice and tilted scanning plane were used to reduce susceptibility-related signal loss at the basal forebrain [25]. Stimuli were displayed on MRI-compatible video goggles (Resonance Technology, Northridge, CA). Each functional run acquired 258 volumes.

Each BOLD time series was motion-corrected, normalized to the MNI (Montreal Neurological Institute) template, and smoothed with a 5-mm kernel using SPM2 (Statistical Parametric Mapping, Welcome Department of Cognitive Neurology, London). After the functional images were filtered with a 128-s high-pass temporal filter, model time courses for each block condition were constructed by convolving a boxcar waveform representing the times of the presentation of each block with the canonical hemodynamic response function offered by SPM2. The functional data were analyzed using a standard GLM.

Statistical tests had two levels, first at single subject level (fixed effects) and then at group level (random effects). For each subject, SPM (T} maps with the following contrasts were created first: high load vs. low load (collapsed across distractor conditions), low load (with distractor vs. without distractor), high load (with distractor vs. without distractor), and [low load (with distractor vs. without distractor) vs. high load (with distractor vs. without distractor)]. Then, these contrast maps were fed into the second level, one-sample t-tests to acquire group means for each contrast. Unless specified otherwise in the result section, we employed FWE cluster-level correction for multiple comparisons of the voxel-wise whole brain analysis throughout this study, and used cluster p<.05 (FWE corrected) in conjunction with voxel height threshold p<.01 to identify significant changes in BOLD signal between task conditions. Marsbar toolbox [26] was used to define significant clusters as functional ROIs and extract percent BOLD signal changes from functional ROIs. The extracted percent changes in signal were used in figures for demonstrating signal changes between task conditions in specified ROIs. No statistical analysis was performed on data extracted from any ROIs to avoid circular analysis.

## Results

### Task Performance

**Figure 2:**
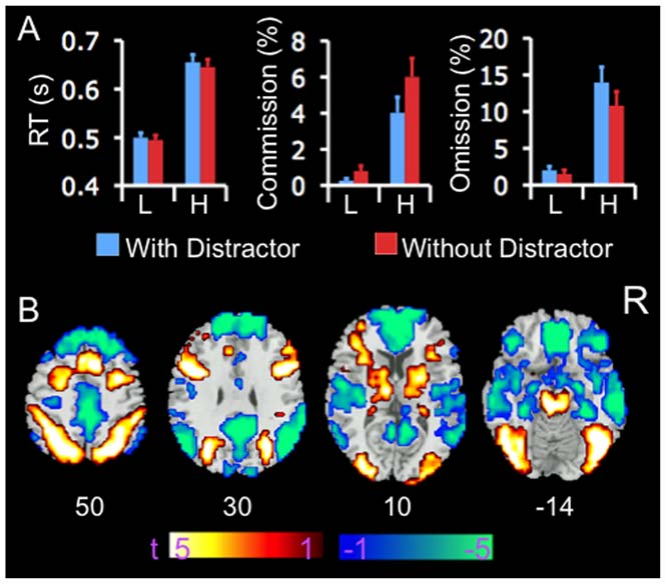
Performance data and BOLD signal changes on the task. A). Error bar indicates standard error of means. Perceptual load showed main effects on commission and omission errors and on RT. Distractors showed significant main effects on commission and omission errors, but only a trend on RT. Perceptual loads and distractors showed a significant two-way interaction with respect to commission errors, and trended towards a significant two-way interaction with respect to omission errors. B) Color on T1 template image from SPM2 indicates significant increases (red color) and decreases (blue color) in BOLD signal in the condition of high relative to low perceptual load, collapsed across distractor conditions. The color bar indicates t value. The number under each brain image indicates the Z coordinate of the image in the MNI (Montreal Neurological Institute) template space. The only voxels displayed on the brain images are those surviving voxel threshold <.01 and cluster level p<.05, FWE corrected for multiple comparisons of voxel-wise whole brain analysis. Abbreviations: L: low load; H: high load; R: right side; RT: reaction time.

Fig. 2A presents the performance data. There was a significant main effect of perceptual load on commission (F (1, 23) = 28.10, p<.001) and omission errors (F (1, 23) = 35.23, p<.001) and on RT (F (1, 23) = 242.07, p<.001). A significant main effect of distractor was observed on commission (F (1, 23) = 13.51, p<.001) and omission errors (F (1, 23) = 6.23, p<.05), and a marginal main effect on RT (F (1, 23) = 4.1, p = .053). A significant two-way interaction was observed between perceptual load and distractor on commission error rates (F (1, 23) = 8.51, p<.01), and a trend towards a two-way interaction on omission error rates (F (1, 23) = 3.41, p = .078). No interaction effect was observed with respect to RT (F (1, 23) = 0.46, p = .50). Post-hoc paired t-test analyses revealed that commission error rates showed a greater decrease in the condition with distractors relative to in the condition without distractors at high task load (t = −3.8, df = 23, p = .001) than at low task load (t = −1.9, df = 23, p = .073). Omission error rates showed a greater increase in the condition with distractors relative to in the condition without distractors at high load (t = 2.3, df = 23, p = .032) than at low task load (t = 1.2, df = 23, p = .23). Distractor-related changes in omission and commission errors were not significantly correlated with each other at either perceptual load. Taken together, these data indicate that the task imposed greater attentional demand at high than low perceptual load conditions, and that distractors significantly decreased and increased commission and omission errors, respectively, at high load, but did not significantly influence either types of errors at low load.

### Load-Related Changes in BOLD Signal

Participants showed increases in BOLD signal in brain regions implicated in attentional processing, including the lateral PFC (LPFC), parietal cortex, occipital cortex including the fusiform gyrus, thalamus, basal ganglia, and midbrain, at high relative to low perceptual load, across distractor conditions (Fig. 2B). They showed decreases in BOLD signal in regions of the default-mode network, including the medial PFC (MPFC), posterior cingulate (PCC)/precuneus, temporoparietal junction (TPJ), and medial temporal lobes including the hippocampus, parahippocampus, and amygdala, bilaterally, at high relative to low load. This pattern of task-related increases and decreases in BOLD signal suggests that the task imposed greater attentional demands during the high load relative to low load condition.

### Distractor-Related Changes in BOLD Signal

#### Low perceptual load

**Figure 3:**
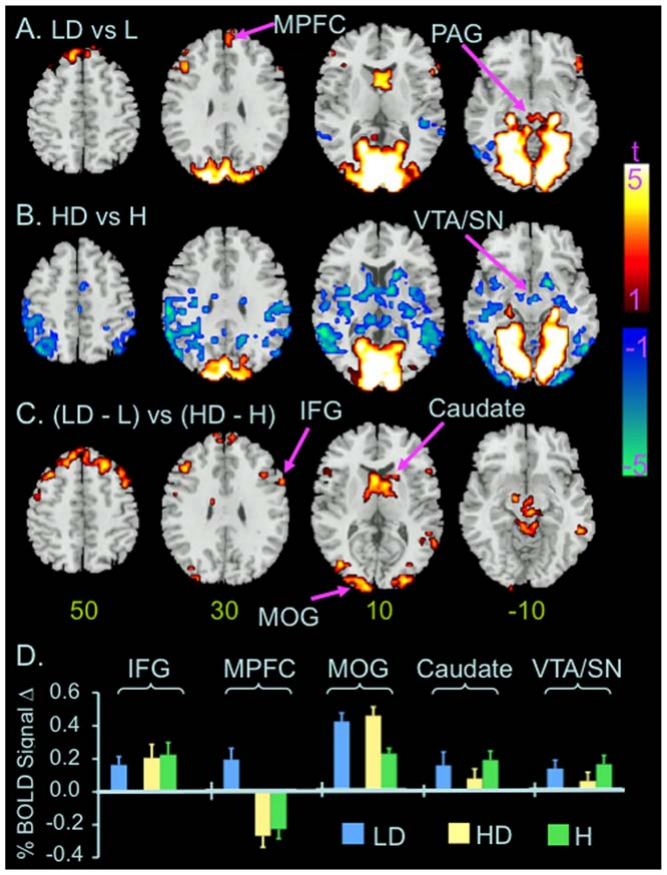
Distractor related changes in BOLD signal at different perceptual load. Color on T1 template images from SPM2 indicates significant distractor-related increases (red color) or decreases (blue color) in BOLD signal in the conditions of low (A) and high perceptual load (B), and differences in distractor-related changes in BOLD signal at low vs. high perceptual load (C). The color bar indicates t value. The number under each brain image at the bottom row indicates the Z coordinate of the brain image in the MNI (Montreal Neurological Institute) template space. The only voxels displayed on the brain images are those surviving voxel threshold <.01 and cluster level p<.05, FWE corrected for multiple comparisons of voxel-wise whole brain analysis. D) Bar graphs show changes in BOLD signal in the labeled ROIs. The level of BOLD signal at the condition of low load without distractor was defined as baseline (0). Error bars indicates standard errors of means. Abbreviation: H: high load without distractor; HD: high load with distractors; IFG: inferior frontal gyrus; L: low load without distractors; LD: low load with distractors; MOG: middle occipital gyrus; MPFC: medial prefrontal cortex; PAG: periaqueductal gray; R: right; SFG: superior frontal gyrus; VTA/SN: ventral tegmental area and adjacent substantia nigra.

Five clusters showed significant increases in BOLD signal in the distractor relative to the no–distractor condition at low perceptual load (Fig. 3A & Table 1). One cluster includes the occipital cortex, cerebellum, lateral geniculate nucleus, parahippocampus, thalamus, and midbrain regions including periaqueductal gray and reticular formation (PAG/RF), bilaterally. The other clusters include the right IFG, left middle frontal gyrus (MFG), bilateral caudate heads and dorsal MPFC. Two clusters, each at the right and left TPJs, showed significant decreases in BOLD signal in the distractor relative to no–distractor condition at low perceptual load (Fig. 3A & Table 1).

**Table 1:** Distractor-related changes in BOLD signal.

					MNI Coordinates		
	L/R	BA	Size (voxels)	Z-Value	X	Y	Z
**Low Load (Distractor – No Distractor)**							
***Positive***							
Occipital Cortex, Parahippocampus	L/R	17, 18, 19, 28	5883	7.5	0	−90	3
Caudate Head	L/R		173	4.2	6	6	12
Dorsal MPFC	L/R	8, 9	176	4.1	−6	45	54
Middle Frontal G.	L	9, 46	93	3.8	−48	30	36
Inferior Frontal G.	R	45, 47	201	3.7	54	21	3
***Negative***							
Superior & Middle Temporal G.	L	19, 22, 37	132	4.1	−45	−66	−3
Superior Temporal G.	R	22, 41	91	3.3	42	−21	3
**High Load (Distractor – No Distractor)**							
***Positive***							
Cuneus, Lingual G., Parahippocampus	L/R	17, 18, 19, 28	4701	7.0	0	−90	3
***Negative***							
Parietal, Temporal, & Occipital Cortices, Insula, PCC/Precuneus, Thalamus, Striatum, VTA/SN, Hippocampus	L/R	1, 3, 7, 18, 19, 20, 21, 22, 29, 30, 31, 37, 39, 40, 41, 42	5173	5.7	−51	−66	0
Dorsal ACC	L/R	23	82	4.0	3	−12	39
Cerebellum	L/R		173	3.6	0	−42	−24
**Low Load (Distractor – No Distractor) vs. High Load (Distractor – No Distractor)**							
***Positive***							
Cuneus, Middle Occipital G.	L	18, 19	291	4.3	−12	−102	15
Caudate, Thalamus, Midbrain	L/R		640	4.2	9	3	18
Superior Frontal & Precentral G.	R	4, 6	138	4.1	9	−12	72
Cuneus, Middle Occipital G	R	18, 19	118	4.1	33	−93	15
Middle Temporal G.	R	22	106	4.0	60	−63	3
Medial & Lateral Superior Frontal G.	L/R	8, 9	416	4.0	21	30	51
Middle & Inferior Frontal G.	L	6, 9	192	3.7	−36	21	51
Inferior Frontal G.	R	9, 45	87	3.6	54	21	3

All clusters in this table were generated at FWE corrected cluster p<.05 with voxel level p<.01. Abbreviations: ACC: anterior cingulate; BA: Brodmann area; G: Gyrus; L: left; MPFC: medial prefrontal cortex; R: right; Size: number of voxels in the cluster; VTA/SN: ventral tegmental area/substantia nigra; Z-value: the Z value of peak voxel in the cluster.

#### High perceptual load

**Figure 4:**
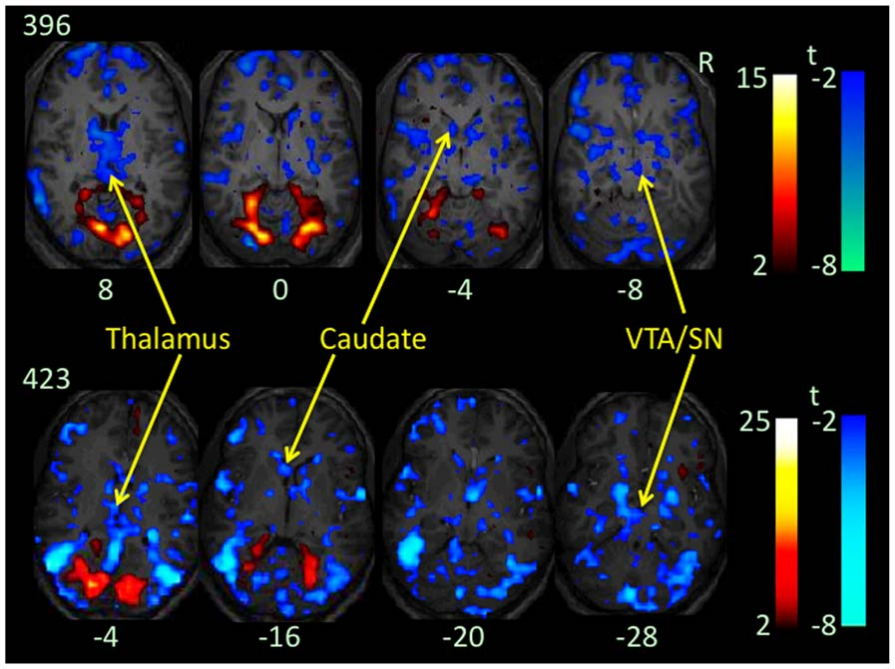
Distractor-related changes in BOLD signal at high load in the native space. Color on T1 anatomical images of two participants (396 and 423) without spatial normalization indicate increases (red color) or decreases (blue color) in BOLD signal at contrast high load (with distractors vs. without distractors). Voxel level threshold is p<.01 without corrections for multiple comparisons of voxel-wise whole brain analysis. The number under each brain image indicates the Z coordinate of the brain image in their original space. Please note decreases in BOLD signal in the caudate head, thalamus, and ventral tegmental area and adjacent substantia nigra (VTA/SN).

One cluster across the occipital cortex including cuneus and lingual gyrus, parahippocampus, and cerebellum, bilaterally, showed significant increases in BOLD signal at the distractor relative to no-distractor condition at high perceptual load (Fig. 3B & Table 1). Three clusters showed decreases in BOLD signal at the distractor relative to no-distractor condition at high perceptual load (Fig. 3B, Fig. 4 & Table 1). One covers extensive regions across the parietal, temporal, and occipital cortices including middle and inferior occipital gyri, posterior insula, thalamus, striatum, and ventral tegmental area and adjacent substantia nigra (VTA/SN), bilaterally. To better localize significant decreases in BOLD signal in this large cluster, we reanalyzed the contrast of high load [with distractors vs. without distractor] using cluster-based FWE corrected p<.05 for multiple comparisons of voxel-wise whole brain analysis in conjunction with voxel high threshold p<.001. This secondary analysis revealed significant decreases in BOLD signal in 10 clusters at the distractor relative to no-distractor condition. These clusters were distributed in the bilateral temporal and occipital cortices and caudate heads, left parietal and insula cortices, and right thalamus and VTA/SN (Table 2).

**Table 2:** Distractor-related decreases in BOLD signal at high load.

					MNI Coordinates		
	L/R	BA	Size (voxels)	Z-Value	X	Y	Z
Superior & Middle Temp G.	L	22, 37	330	5.7	−51	−66	0
Superior & Middle Temp G.	R	22, 37	214	5.0	57	−66	0
Putamen & Caudate Head, Thalamus, VTA/SN	R		121	5.0	15	6	−9
Putamen & Caudate Head	L		54	4.1	−12	0	15
Middle & Inferior Occipital G.	R	18	55	5.0	27	−96	−3
Middle & Inferior Occipital G.	L	18	38	4.7	−33	−99	3
Inferior Parietal Lobule	L	40	119	4.8	−45	−33	21
Inferior Parietal Lobule	L	3, 40	44	3.9	−30	−36	33
Superior Parietal Lobule	L	7	54	4.2	−42	−66	51
Insula	L	13	54	4.2	−45	−3	0

All clusters in this table were generated at FWE corrected cluster p<.05 with voxel level p<.001. Abbreviations: G: Gyrus; L: left; R: right; Size: number of voxels in the cluster; VTA/SN: ventral tegmental area/substantia nigra; Z-value: the Z value of peak voxel in the cluster.

#### Low vs. high perceptual load

Eight clusters showed significant differences in distractor-related changes in BOLD signal at low vs. high perceptual load as assessed by the contrast [low load (with distractor vs. without distractor) vs. high load (with distractor vs. without distractor)] (Fig. 3C & Table 1). These clusters include the IFG, MPFC, occipital cortex including the cuneus and middle occipital gyrus, caudate head, thalamus, VTA/SN, and PAG/RF, bilaterally. Inspection of percent signal changes extracted from functional ROIs showed that these differences were due to either a greater distractor-related increase (e.g., occipital cortex) or a smaller distractor-related decrease (e.g., bilateral TPJs) in BOLD signal at low than high perceptual load, respectively, or distractor-related increase vs. decrease in BOLD signal at low vs. high perceptual load (e.g., MPFC, caudate head, VTA/SN, respectively, Fig. 3D).

## Discussion

The main aim of this study was to test our hypothesis that distractors would induce greater activation in frontostriatal regions at a low relative to a high perceptual load of a central task. Our main findings include: 1) distractors induced a greater decrease in commission errors and a trend towards a greater increase in omission errors at high as compared to low perceptual load; 2) distractors induced an increase in activation in the occipital cortex and parahippocampus at both low and high loads, and the increases were greater at low than high load, consistent with prior work; 3) distractors induced an increase in activation in multiple brain regions including the IFG, caudate head, and midbrain PAG/RF at low load; and, 4) distractors induced a decrease in activation in the striatum, midbrain VTA/SN, and multiple sensory cortices at high load. These findings support our prediction that distractors would induce a greater increase in activation in frontostriatal regions at low relative to high load.

### Task Performance

In this study, distractors showed a marginal main effect on RT. This effect is weaker than the significant distractor-related RT increases typically reported by prior load theory studies [5], [8]. Distractors used in the current study did not share features with targets and would likely not induce responses when they were perceived. Therefore they had a “neutral” effect on target responses and a weak effect on RT. Furthermore, distractors appeared in every stimulus during the “distractor blocks,” so they may have been predictable and thus potentially less distracting [27]–[29]. Indeed, the current weak effect of distractors on RT is consistent with the findings of no distractor-related significant RT increases reported by previous studies using block design and “neutral” distractors [10], [15]. Notably, Forster and Lavie reported a significant effect of neutral distractors on RT in one study [30]. However, the neutral distractors used in that study were very salient and their appearances were unpredictable. These features of the neutral distractors might contribute to their significant effects on RT.

In the current study, distractors were associated with increased omission errors and decreased commission errors. The distractor-induced opposite changes in the errors of omission and commission are consistent with a previous hypothesis that they are associated with different central processes, i.e., processes related to attention vs. impulsivity [21]–[24]. Previous studies reported differential effects of distractors on commission and omission errors [21], [31], [32]. For example, increasing distractor saliency from just above perceptual threshold improved the accuracy of healthy participants on a color discrimination task [33]. While healthy participants performed visual vigilance tasks, auditory noise decreased omission error only in one study [21], but decreased commission error only in another study [34]. Therefore, the differential effects of neutral distractors on omission and commission errors observed in the current study were in line with previously reported differential effects of distractors on errors of omission and commission [21], [34].

The distractor-related changes in omission and commission errors did not correlate with each other at either perceptual load. The lack of such a correlation suggests that the opposite changes in the two types of errors are probably not due to a tradeoff between them. The significant two-way interaction between perceptual load and distractors on commission error suggests that the effects of distractors on commission errors are dependent on perceptual load, and probably reflect different neural correlates of suppressing distractor interference at different perceptual loads (see below). However, we cannot exclude the potential contribution of a “floor effect” with respect to the two-way interaction, because the commission error rate was very low in the condition of low perceptual load without distractor (mean = 0.8%, SD = 1.4) and therefore might not allow assessment of further decreases.

### Neural Correlates of Suppressing Distractor Interference at Low Perceptual Load

Throughout this paper we use the phrases “distractor-related activation”, “distractor-related deactivation”, and “distractor-related changes in activation” to refer to the observed statistically significant increases, decreases, and differences, respectively, in BOLD signal between conditions with and without distractors at each level of perceptual load. By using these phrases we do not imply that these changes in BOLD signal are involved *solely* in distractor processing and not involved in processing of relevant stimuli (e.g., faces). The current design did not allow us to isolate BOLD signal in the frontostriatal circuits associated with distractors only (e.g., scene pictures) from BOLD signal associated with relevant stimuli only, or with both distractors and relevant stimuli.

The participants in this study showed distractor-related increases in activation in the visual cortex, parahippocampus and frontostriatal regions including IFG and caudate heads at low perceptual load. The distractor-related activation in the parahippocampus is consistent with a previous finding that the parahippocampus is specifically sensitive to stimuli of houses and/or scenes [19]. In frontostriatal circuits, the striatum receives diffuse inputs from the cortex and projects mainly to the PFC through the pallidum and thalamus [35], [36]. These circuits are implicated in differentiating targets from distractors and in selecting appropriate responses [37]–[42]. The distractor-related activation increases in the bilateral caudate heads, right IFG and left middle frontal gyrus (MFG) at low load probably reflect increased processing of sensory inputs and are consistent with the prediction of load theory that distractor interference is resolved at later stages of central processing at low load [8], [43]. The increased activation could be involved in discriminating distractors (scenes) from relevant stimuli (e.g., target and non-target faces), rejecting distractors, and/or selecting responses; however, we cannot isolate activation associated with each of these cognitive processes in this study.

The MPFC, a major component of the brain default-mode network, often shows relative deactivation while healthy participants perform cognitively demanding tasks relative to a control condition [44], [45]. However, it is also implicated in arousal and increases activation while healthy participants perform tasks with low cognitive demand relative to a control condition [46]–[48]. Similarly, the midbrain PAG/RF is implicated in arousal and has been reported to activate while participants perform a demanding cognitive task relative to a less demanding control condition [49]. Therefore, current distractor-related activation increases in the MPFC and PAG/RF at low load suggest that participants may have increased their arousal and attention to suppress distractor interference. This interpretation is consistent with previously observed distractor-related increases in arousal and attention while healthy participants performed cognitive tasks [50], [51].

The TPJs are often relatively deactivated while healthy subjects perform cognitive tasks, and this deactivation has been proposed to reflect suppression of distractor processing in the brain [52], [53]. Based on this notion, the distractor-related TPJ deactivation at low load in the present study might reflect distractor filtering. The distractors filtered in the TPJs, however, may include distractors other than task-introduced scene distractors. The TPJs have been implicated not only in visual attention [54], but also in other cognitive processes including self-attribution, embodiment, empathy, and mentalization [55]–[58]. These cognitive processes (e.g., self-attribution) are irrelevant to target identification while participants are on-task, and therefore the information processed in them should be suppressed.

The above-discussed findings together suggest that three sets of different neural events operate to prevent interference from task-introduced distractors at low perceptual load. The first may involve increasing activity in the midbrain PAG/FR and MPFC to increase arousal and attention to external stimuli; the second may involve increasing activity in the visual cortex and frontostriatal circuits, which may reflect increased processing of sensory inputs at both early and late stages of central processing; and the third may involve decreasing activity in the TPJs to filter distractors.

### Neural Correlates of Suppressing Distractor Interference at High Perceptual Load

The cuneus, lingual gyrus, and parahippocampus showed increased activation in the condition with distractors relative to without distractors at high load. This increase could be accounted for by distractor-related processing in the visual cortices. Furthermore, distractors induced diminished activation (i.e., deactivation) in the middle and inferior occipital gyri at high load. In this instance, “deactivation” refers to the finding of reduced BOLD signal in the condition with distractors relative to without distractors at high perceptual load. This pattern of activation and deactivation together results in smaller distractor-related net increases in activation in the cuneus and middle occipital gyri at high relative to low load. These data are consistent with findings of prior studies of the load theory that the occipital cortex shows smaller distractor-related increases in activation at high relative to low load.

The distractors at high load were also associated with deactivation in the parietal, temporal, and posterior insular cortices, which receive somatosensory, auditory, and visceral sensory inputs, respectively [59]–[64]. These sensory inputs are irrelevant to target identification and thus are distractors while participants are on-task. The extensive deactivation of these sensory cortices suggests that the task-introduced visual distractors lead to inhibition of central processing of not only irrelevant visual inputs, but also inputs in other sensory modalities. This finding of cross-modality inhibition is consistent with cross-modality deactivation observed in several fMRI studies [13], [14], [65]–[67].

At high load, distractors were not associated with increases in activation in frontostriatal regions, but with relative decreases in activation in the caudate heads and thalamus. This finding is consistent with our prediction that distractors will evoke a smaller increase in activation in the frontostriatal circuits at high relative to low load. The precise neural mechanisms underlying distractor-induced deactivation in the caudate, thalamus, and extensive sensory cortices (e.g., parietal & temporal cortices) at high load are not clear at present. They might relate to top-town attentional control, which might inhibit the processing of irrelevant information in the sensory cortices and thus prevent it from reaching frontostriatal regions when most or all processing resources of the frontostriatal regions are consumed by high-demanding central tasks.

An unexpected finding of this study was the distractor-related deactivation in the VTA/SN at high load. Previous studies reported that the level of BOLD signal change in the VTA/SN was positively correlated with levels of their DA release in the frontostriatal circuits while healthy participants performed cognitive tasks [68]–[70]. Therefore, the reduced BOLD signal in the VTA/SN might reflect a decrease in DA release in the frontostriatal circuitry in the condition with distractor relative to that without distractors at high load. Decreased DA level in the frontostriatal circuits would be expected to decrease their neuronal responsiveness to sensory inputs [37], [39]–[42], [71]–[73]. Therefore, distractor-related decreases in activation in the VTA/SN at high load might contribute to distractor-related deactivation in the frontostriatal circuits. Effective connectivity between VTA/SN and the frontostriatal circuits should be assessed in future studies specifically designed for such a purpose.

In this study, participants needed to remember more features when they tried to identify targets by searching for a junction of features in the condition of high load relative to when they tried to identify targets by searching for one feature in the condition of low load. Therefore, working memory load was greater in the condition of high relative to low load. It has been hypothesized that increasing working memory load would consume increasingly large resources of cognitive control and reduce inhibition of distractor processing [6], [8]. Consistent with this hypothesis, several studies found that increasing working memory load of a secondary task would increase distractor interference [74]–[77]. However, several other fMRI studies reported that increasing working memory load of the main task increased distractor inhibition in the sensory cortex [9], [78]–[80]. Therefore, it seems to us that the effect of working memory load on distractor inhibition depends on its association with the main and secondary task. Here, we propose that working memory load will increase distractor inhibition if it associates with the main task, and that working memory load will decrease distractor inhibition if it associates with a secondary task. In the current study, the increased working memory load at high load condition associated with the main task and therefore it probably increased distractor inhibition. Therefore, the current findings of reduced distractor processing in the visual cortex and frontostriatal circuits at high relative to low load condition were probably due to the joint effect of increased perception load and working memory load.

### Limitations

A potential weakness of this study is its block design, which prevents the isolation of brain activity associated with performance errors, particularly since error rates are greater at high relative to low task load. However, block designs have been frequently used in studies investigating the neural substrates of attention, including within the context of the load theory [7], [9]–[15]. Furthermore, we do not believe the observed differences in distractor-related brain activity at different task loads are likely to be accounted for by different error rates as there is no significant difference in total error rates (i.e., sum of commission and omission errors) between conditions with vs. without distractors at either task load. Therefore, significant changes in BOLD signal detected by contrast (condition with distractor vs. condition without distractor) at each task load are likely not to be substantially attributable to differences in performance errors between task conditions. A second limitation involves the use of “neutral” distractors. This feature may contribute to smaller effects of distractors on RT in this study relative to those of previous studies investigating processes related to load theory.

### Conclusion

Findings from this study indicate that distractor-related activation is greater not only in the sensory cortices, but also in frontostriatal circuits at low relative to high perceptual load. At low perceptual load, the neural mechanisms of inhibiting distractor interference may include increasing arousal by increasing activation in the midbrain PAG/FR and MPFC, filtering distractors by decreasing activation in the TPJ, and rejecting distractors and/or selecting appropriate responses by increasing activation in frontostriatal circuitry. At high perceptual load, increased top-down attentional control appears to contribute to the inhibition of distractor interference, as suggested by distractor-related concurrent deactivation in the frontostriatal circuits, midbrain VTA/SN, and extensive sensory cortices. Therefore, the midbrain PAG/FR and VTA/SN and frontostriatal circuits may play different roles in inhibiting distractor interference at different perceptual demands of central tasks.

## References

[1] Treisman A, Geffen G (1967). Selective attention: perception or response?. Q J Exp Psychol.

[2] Deutsch JA, Deutsch D (1963). Some theoretical considerations.. Psychol Rev.

[3] Deutsch JA, Deutsch D, Lindsay PH, Treisman AM (1967). Comments and reply on “Selective attention: perception or response?. Q J Exp Psychol.

[4] Duncan J (1980). The locus of interference in the perception of simultaneous stimuli.. Psychol Rev.

[5] Lavie N, Tsal Y (1994). Perceptual load as a major determinant of the locus of selection in visual attention.. PerceptPsychophys.

[6] Lavie N (2005). Distracted and confused?: selective attention under load.. Trends Cogn Sci.

[7] Rees G, Frackowiak R, Frith C (1997). Two modulatory effects of attention that mediate object categorization in human cortex.. Science.

[8] Lavie N, Hirst A, de Fockert JW, Viding E (2004). Load theory of selective attention and cognitive control.. J Exp Psychol Gen.

[9] Pinsk MA, Doniger GM, Kastner S (2004). Push-pull mechanism of selective attention in human extrastriate cortex.. J Neurophysiol.

[10] Schwartz S, Vuilleumier P, Hutton C, Maravita A, Dolan RJ (2005). Attentional load and sensory competition in human vision: modulation of fMRI responses by load at fixation during task-irrelevant stimulation in the peripheral visual field.. Cereb Cortex.

[11] Yi DJ, Woodman GF, Widders D, Marois R, Chun MM (2004). Neural fate of ignored stimuli: dissociable effects of perceptual and working memory load.. Nat Neurosci.

[12] Bahrami B, Lavie N, Rees G (2007). Attentional load modulates responses of human primary visual cortex to invisible stimuli.. Curr Biol.

[13] Berman RA, Colby CL (2002). Auditory and visual attention modulate motion processing in area MT+.. Brain Res Cogn Brain Res.

[14] Klemen J, Buchel C, Rose M (2009). Perceptual load interacts with stimulus processing across sensory modalities.. Eur J Neurosci.

[15] Yucel G, Petty C, McCarthy G, Belger A (2005). Graded visual attention modulates brain responses evoked by task-irrelevant auditory pitch changes.. J Cogn Neurosci.

[16] Boulougouris V, Tsaltas E (2008). Serotonergic and dopaminergic modulation of attentional processes.. Prog Brain Res.

[17] Chudasama Y, Robbins TW (2006). Functions of frontostriatal systems in cognition: comparative neuropsychopharmacological studies in rats, monkeys and humans.. Biol Psychol.

[18] Owen AM (2004). Cognitive dysfunction in Parkinson's disease: the role of frontostriatal circuitry.. Neuroscientist.

[19] Epstein R, Kanwisher N (1998). A cortical representation of the local visual environment.. Nature.

[20] Kanwisher N, Yovel G (2006). The fusiform face area: a cortical region specialized for the perception of faces.. Philos Trans R Soc Lond B Biol Sci.

[21] Ballard JC (1996). Computerized assessment of sustained attention: a review of factors affecting vigilance performance.. J Clin Exp Neuropsychol.

[22] Conners CK, Epstein JN, Angold A, Klaric J (2003). Continuous performance test performance in a normative epidemiological sample.. J Abnorm Child Psychol.

[23] Halperin JM (1991). The clinical assessment of attention.. International Journal of Neuroscience.

[24] Moeller FG, Hasan KM, Steinberg JL, Kramer LA, Dougherty DM (2005). Reduced anterior corpus callosum white matter integrity is related to increased impulsivity and reduced discriminability in cocaine-dependent subjects: diffusion tensor imaging.. Neuropsychopharmacology.

[25] Deichmann R, Gottfried JA, Hutton C, Turner R (2003). Optimized EPI for fMRI studies of the orbitofrontal cortex.. Neuroimage.

[26] Brett M, Anton JL, Valabregue R, Poline JB (2002). Region of interest analysis using an SPM toolbox.. Neuroimage.

[27] Woods DL, Alain C, Covarrubias D, Zaidel O (1993). Frequency-related differences in the speed of human auditory processing.. Hear Res.

[28] Floden D, Vallesi A, Stuss DT (2010). Task Context and Frontal Lobe Activation in the Stroop Task.. J Cogn Neurosci.

[29] Kerns JG, Cohen JD, MacDonald AW, Cho RY, Stenger VA (2004). Anterior cingulate conflict monitoring and adjustments in control.. Science.

[30] Forster S, Lavie N (2007). High perceptual load makes everybody equal: eliminating individual differences in distractibility with load.. Psychol Sci.

[31] Holding DH, Baker MA (1987). Toward meaningful noise research.. J Gen Psychol.

[32] Loeb M (1981). The present state of research on the effects of noise: are we asking the right questions?. J Aud Res.

[33] Sheremata S, Sakagami M (2006). Increasing distractor strength improves accuracy.. Percept Mot Skills.

[34] Davies DR, Hockey GR, Taylor A (1969). Varied auditory stimulation, temperament differences and vigilance performance.. Br J Psychol.

[35] Alexander GE, Crutcher MD (1990). Functional architecture of basal ganglia circuits: Neural substrates of parallel processing.. Trends in Neurosciences.

[36] Herrero MT, Barcia C, Navarro JM (2002). Functional anatomy of thalamus and basal ganglia.. Childs Nerv Syst.

[37] Kropotov JD, Etlinger SC (1999). Selection of actions in the basal ganglia-thalamocortical circuits: review and model.. Int J Psychophysiol.

[38] Middleton FA, Strick PL (2000). Basal ganglia output and cognition: evidence from anatomical, behavioral, and clinical studies.. Brain Cogn.

[39] Goto Y, Otani S, Grace AA (2007). The Yin and Yang of dopamine release: a new perspective.. Neuropharmacology.

[40] Lapish CC, Kroener S, Durstewitz D, Lavin A, Seamans JK (2007). The ability of the mesocortical dopamine system to operate in distinct temporal modes.. Psychopharmacology (Berl).

[41] McNab F, Klingberg T (2008). Prefrontal cortex and basal ganglia control access to working memory.. Nat Neurosci.

[42] Seamans JK, Yang CR (2004). The principal features and mechanisms of dopamine modulation in the prefrontal cortex.. Prog Neurobiol.

[43] Lavie N (1995). Perceptual load as a necessary condition for selective attention.. J Exp Psychol Hum Percept Perform.

[44] Sonuga-Barke EJ, Castellanos FX (2007). Spontaneous attentional fluctuations in impaired states and pathological conditions: a neurobiological hypothesis.. Neurosci Biobehav Rev.

[45] Raichle ME, MacLeod AM, Snyder AZ, Powers WJ, Gusnard DA (2001). A default mode of brain function.. Proc Natl Acad Sci U S A.

[46] Gilbert SJ, Simons JS, Frith CD, Burgess PW (2006). Performance-related activity in medial rostral prefrontal cortex (area 10) during low-demand tasks.. J Exp Psychol Hum Percept Perform.

[47] Gilbert SJ, Spengler S, Simons JS, Steele JD, Lawrie SM (2006). Functional specialization within rostral prefrontal cortex (area 10): a meta-analysis.. J Cogn Neurosci.

[48] Gilbert SJ, Spengler S, Simons JS, Frith CD, Burgess PW (2006). Differential functions of lateral and medial rostral prefrontal cortex (area 10) revealed by brain-behavior associations.. Cereb Cortex.

[49] Kinomura S, Larsson J, Gulyas B, Roland PE (1996). Activation by attention of the human reticular formation and thalamic intralaminar nuclei.. Science.

[50] Davies DR, Jones DM (1975). The effects of noise and incentives upon attention in short-term memory.. Br J Psychol.

[51] Gulian E (1970). Effects of noise on arousal level in auditory vigilance.. Acta Psychol (Amst).

[52] Shulman GL, McAvoy MP, Cowan MC, Astafiev SV, Tansy AP (2003). Quantitative analysis of attention and detection signals during visual search.. J Neurophysiol.

[53] Shulman GL, Astafiev SV, McAvoy MP, d'Avossa G, Corbetta M (2007). Right TPJ deactivation during visual search: functional significance and support for a filter hypothesis.. Cereb Cortex.

[54] Corbetta M, Shulman GL (2002). Control of goal-directed and stimulus-driven attention in the brain.. Nat Rev Neurosci.

[55] Decety J, Lamm C (2007). The role of the right temporoparietal junction in social interaction: how low-level computational processes contribute to meta-cognition.. Neuroscientist.

[56] Lopez C, Halje P, Blanke O (2008). Body ownership and embodiment: vestibular and multisensory mechanisms.. Neurophysiol Clin.

[57] Newman-Norlund RD, Bosga J, Meulenbroek RG, Bekkering H (2008). Anatomical substrates of cooperative joint-action in a continuous motor task: virtual lifting and balancing.. Neuroimage.

[58] Schulte-Ruther M, Markowitsch HJ, Shah NJ, Fink GR, Piefke M (2008). Gender differences in brain networks supporting empathy.. Neuroimage.

[59] Kostopoulos P, Albanese MC, Petrides M (2007). Ventrolateral prefrontal cortex and tactile memory disambiguation in the human brain.. Proc Natl Acad Sci U S A.

[60] Nagai M, Kishi K, Kato S (2007). Insular cortex and neuropsychiatric disorders: a review of recent literature.. Eur Psychiatry.

[61] Morosan P, Rademacher J, Schleicher A, Amunts K, Schormann T (2001). Human primary auditory cortex: cytoarchitectonic subdivisions and mapping into a spatial reference system.. Neuroimage.

[62] Abdul-Kareem IA, Sluming V (2008). Heschl gyrus and its included primary auditory cortex: structural MRI studies in healthy and diseased subjects.. J Magn Reson Imaging.

[63] Wall JT, Xu J, Wang X (2002). Human brain plasticity: an emerging view of the multiple substrates and mechanisms that cause cortical changes and related sensory dysfunctions after injuries of sensory inputs from the body.. Brain Res Brain Res Rev.

[64] Shelley BP, Trimble MR (2004). The insular lobe of Reil–its anatamico-functional, behavioural and neuropsychiatric attributes in humans–a review.. World J Biol Psychiatry.

[65] Ciaramitaro VM, Buracas GT, Boynton GM (2007). Spatial and cross-modal attention alter responses to unattended sensory information in early visual and auditory human cortex.. J Neurophysiol.

[66] Hairston WD, Hodges DA, Casanova R, Hayasaka S, Kraft R (2008). Closing the mind's eye: deactivation of visual cortex related to auditory task difficulty.. Neuroreport.

[67] Mozolic JL, Joyner D, Hugenschmidt CE, Peiffer AM, Kraft RA (2008). Cross-modal deactivations during modality-specific selective attention.. BMC Neurol.

[68] D'Ardenne K, McClure SM, Nystrom LE, Cohen JD (2008). BOLD responses reflecting dopaminergic signals in the human ventral tegmental area.. Science.

[69] Nyberg L, Andersson M, Forsgren L, Jakobsson-Mo S, Larsson A (2009). Striatal dopamine D2 binding is related to frontal BOLD response during updating of long-term memory representations.. Neuroimage.

[70] Schott BH, Minuzzi L, Krebs RM, Elmenhorst D, Lang M (2008). Mesolimbic functional magnetic resonance imaging activations during reward anticipation correlate with reward-related ventral striatal dopamine release.. J Neurosci.

[71] Kropotov JD, Etlinger SC, Ponomarev VA, Kuznetzov MA, Trofimova LG (1992). Event-related neuronal responses in the human strio-pallido-thalamic system. II. Cognitive functions.. Electroencephalogr Clin Neurophysiol.

[72] Sil'kis IG (2007). The contribution of synaptic plasticity in the basal ganglia to the processing of visual information.. Neurosci Behav Physiol.

[73] Williams GV, Castner SA (2006). Under the curve: critical issues for elucidating D1 receptor function in working memory.. Neuroscience.

[74] de Fockert JW, Rees G, Frith CD, Lavie N (2001). The role of working memory in visual selective attention.. Science.

[75] Dalton P, Santangelo V, Spence C (2009). The role of working memory in auditory selective attention.. Q J Exp Psychol (Colchester).

[76] Kelley TA, Lavie N (2010). Working Memory Load Modulates Distractor Competition in Primary Visual Cortex.. Cereb Cortex.

[77] Lavie N, De Fockert J (2005). The role of working memory in attentional capture.. Psychon Bull Rev.

[78] Klemen J, Buchel C, Buhler M, Menz MM, Rose M (2010). Auditory working memory load impairs visual ventral stream processing: toward a unified model of attentional load.. J Cogn Neurosci.

[79] Rose M, Schmid C, Winzen A, Sommer T, Buchel C (2005). The functional and temporal characteristics of top-down modulation in visual selection.. Cereb Cortex.

[80] Bingel U, Rose M, Glascher J, Buchel C (2007). fMRI reveals how pain modulates visual object processing in the ventral visual stream.. Neuron.

